# The “Double-Edged Sword” of Neutrophils in Trauma: From Pathophysiological Mechanisms to Precision Immunomodulation Strategies

**DOI:** 10.3390/ijms27188091

**Published:** 2026-09-11

**Authors:** Chaoyao Hou, Zhaofeng Kang, Shuang Qin, Zhanfei Li, Xijie Dong

**Affiliations:** 1 Trauma Center, Department of Emergency and Traumatic Surgery, Tongji Hospital, Tongji Medical College, Huazhong University of Science and Technology, Wuhan 430030, China; m202376630@hust.edu.cn (C.H.); d202582875@hust.edu.cn (Z.K.); lezhfei@tjh.tjmu.edu.cn (Z.L.); 2 Department of Radiation Oncology, Hubei Cancer Hospital, Tongji Medical College, Huazhong University of Science and Technology, Wuhan 430030, China; dr.qinshuang@outlook.com

**Keywords:** neutrophils, trauma, neutrophil extracellular traps, immunomodulation, systemic inflammation

## Abstract

Neutrophils occupy a central yet paradoxical position in post-trauma immunity, functioning as both sentinels of host defense and mediators of tissue damage. This review comprehensively examines the spatiotemporal dynamics of neutrophil biology after trauma, ranging from recruitment and effector functions to diverse cell death modalities. We detail how aberrant activation mechanisms, specifically oxidative burst, protease release, and neutrophil extracellular trap formation fuel systemic inflammation, immunothrombosis, and remote organ injury. We also discuss the neutrophil dysfunction-induced immunosuppression and barriers to tissue regeneration. Mechanistically, we dissect the regulatory networks involving NF-κB/MAPK signaling, non-coding RNAs, extracellular vesicles, and metabolic reprogramming. On the clinical front, we evaluate emerging biomarkers and precision therapeutics, such as CXCR2 antagonists and neutrophil extracellular traps inhibitors, designed to recalibrate rather than ablate neutrophil function to balance host defense and tissue repair. We conclude by outlining a future roadmap that leverages single-cell multi-omics and AI to resolve neutrophil heterogeneity and advance personalized interventions for trauma patients.

## 1. Introduction

Trauma, a profound global public health challenge, imposes an enormous socioeconomic burden through its high rates of mortality and persistent disability. Beyond the initial physical insult, severe trauma unleashes a complex pathophysiological cascade centered on a profound dysregulation of the host immune system. While a well-regulated inflammatory response is essential for clearing necrotic tissue and preventing pathogen invasion, this protective mechanism frequently degenerates into an uncontrolled, destructive inflammatory “storm”. This hyper-inflammation drives secondary tissue damage, culminating in multiple organ dysfunction syndrome (MODS) and death. Consequently, deciphering the mechanisms of this post-traumatic immune dysregulation and identifying strategies to guide the response from tissue destruction toward repair represent a critical scientific and clinical challenge. 

Neutrophils, the most abundant leukocytes (50–70%) in human circulation [1], are a critical component of the innate immune system. Under physiological conditions, they are rapidly recruited to sites of injury [2], where they eliminate pathogens and debris through a canonical armamentarium: releasing reactive oxygen species (ROS), secreting proteases, and forming neutrophil extracellular traps (NETs) [3,4]. However, the profound systemic stress of severe trauma induces marked phenotypic and functional alterations in this lineage [5,6]. These hyper-activated neutrophils, while essential for host defense, also release an excessive and indiscriminate barrage of inflammatory mediators and cytotoxic molecules. This activity causes severe “bystander” damage to host tissues [7], exacerbating the systemic inflammatory storm that drives organ failure. This phenomenon highlights the profound dichotomy of neutrophil function in trauma, which positions them as a central nexus in the pathophysiology of post-traumatic immune dysregulation.

An increasing body of clinical and experimental evidence indicates that aberrant neutrophil activation is a key driver of secondary organ injury and adverse long-term outcomes following trauma [8]. Although previous reviews have summarized individual aspects of neutrophil biology during acute inflammation, a comprehensive review that systematically integrates the spatiotemporal dynamics, dual functions, multilayered regulatory networks, and translational implications of neutrophils in the context of trauma remains lacking. This review addresses this gap by systematically outlining the complete biological trajectory of neutrophils following trauma, from their early recruitment and effector activation to programmed cell death and resolution of inflammation. Specifically, this review has three objectives: first, to elucidate the pathophysiological mechanisms by which neutrophils simultaneously mediate host defense and tissue injury after trauma; second, to summarize recent advances in transcriptional, epigenetic, metabolic, and intercellular regulatory networks that govern neutrophil function; and third, to evaluate the translational potential of neutrophil-targeted clinical biomarkers and precision immunotherapeutic strategies.

This review is organized along a logical continuum from basic biology to clinical translation. First, we outline the dynamic evolution of neutrophils following trauma, including their recruitment, activation, and death and clearance. We then dissect the pathological mechanisms through which neutrophils drive infection, distant organ injury, and impaired regeneration via inflammatory amplification, immunothrombosis, and suppression of tissue repair. Next, we focus on regulatory networks involving signal transduction, epigenetic modification, metabolic reprogramming, and intercellular communication. Building on these mechanisms, we evaluate the biomarker potential of neutrophils and current strategies for therapeutic targeting. Finally, we summarize the major gaps in current research and discuss the prospects of integrating single-cell multi-omics with artificial intelligence to characterize neutrophil heterogeneity and advance precision immunomodulatory interventions.

We acknowledge several limitations of this review. Our discussion primarily focuses on severe sterile trauma in adults and does not extensively address burn injuries, pediatric trauma, or trauma complicated by infection, which may involve distinct patterns of neutrophil responses. In addition, owing to space constraints, we do not provide an in-depth discussion of neutrophil heterogeneity across individual organ systems, instead focusing on shared systemic mechanisms. These gaps may serve as important directions for future dedicated reviews.

## 2. Dynamic Biological Processes and Regulatory Mechanisms of Neutrophils Following Trauma

### 2.1. Early Recruitment and Regulation of Neutrophils Post-Trauma

Within minutes of a traumatic insult, a highly orchestrated neutrophil recruitment program is initiated, rapidly establishing them as the dominant immune cell population at the injury site, often peaking within twelve hours [2]. This process is driven by a complex symphony of inflammatory signals and dynamic interactions with the vascular endothelium, ensuring precise migratory fidelity from the circulation into damaged tissue.

The initial “call-to-arms” stems from damage-associated molecular patterns (DAMPs) liberated by injured host cells and pathogen-associated molecular patterns (PAMPs) from invading microbes. These alarmins activate tissue-resident macrophages and endothelial cells, compelling them to release a torrent of chemokines and cytokines [9,10]. This establishes a potent chemoattractant gradient that governs neutrophil migration. Key mediators include interleukin-8 (IL-8/CXCL8), which binds CXCR1/2 receptors on neutrophils to directly induce migration and activation [11,12]; the complement fragment C5a, a potent chemoattractant that also enhances vascular permeability and upregulates endothelial adhesion molecules [13,14]; and leukotriene B4 (LTB4), which effectively amplifies the inflammatory signal [15]. Notably, early arriving neutrophils themselves release LTB4, initiating a positive feedback loop that attracts “swarms” of additional neutrophils [16], exponentially expanding the local cellular infiltrate. 

Neutrophil traversal of the vascular barrier follows the canonical multistep adhesion cascade: rolling, firm adhesion, and transendothelial migration [17]. First, transient, low-affinity interactions between neutrophil ligands (e.g., PSGL-1) and upregulated P- and E-selectins on activated endothelium cause the neutrophils to “roll” along the vessel wall [18,19]. Upon encountering local chemokines (e.g., IL-8), neutrophil chemokine receptors trigger “inside-out” signaling, converting surface β2 integrins (like LFA-1 and Mac-1) from a low- to a high-affinity state. This conformational change facilitates firm binding to endothelial-expressed ICAM-1, leading to neutrophil arrest [20,21]. Finally, neutrophils extend pseudopods and traverse endothelial junctions (diapedesis), migrating along the established chemokine gradient toward the inflammatory core [17,22]. Clinical studies have demonstrated that sustained high expression of neutrophil adhesion molecules (e.g., CD11b/CD18) correlates with uncontrolled inflammation and poor outcomes, underscoring the pathological significance of this process [23]. Consequently, targeting the chemokine receptors or adhesion molecules that mediate this cascade has emerged as a promising strategy to mitigate excessive inflammation and secondary tissue damage.

### 2.2. Activation and Effector Functions of Neutrophils

Once recruited, neutrophils are rapidly activated, unleashing a diverse array of effector functions with a pronounced “double-edged sword” character: they are indispensable for host defense yet capable of initiating profound tissue damage.

Hallmark events of activation include the oxidative burst and protease release [3]. The oxidative burst is driven by the assembly and activation of the NADPH oxidase complex, generating vast quantities of superoxide and its derivatives, collectively termed reactive oxygen species [24]. While physiologically confined to the phagosome as a potent microbicidal weapon, the intense activation during severe trauma leads to extracellular ROS “spillover” [25,26], which causes indiscriminate bystander damage via lipid peroxidation, protein denaturation, and DNA damage to adjacent healthy tissues. 

Concurrently, activated neutrophils undergo degranulation, releasing a potent arsenal of proteases. Among these, neutrophil elastase (NE) is exceptionally destructive. NE can degrade key extracellular matrix (ECM) components like elastin and collagen [4], compromising tissue integrity. It also degrades pulmonary surfactant and coagulation factors, directly impairing organ function. Furthermore, NE perpetuates inflammation by stimulating endothelial cells to release more IL-8 [27] and upregulate ICAM-1 [28], thereby attracting and activating more neutrophils in a vicious, pro-inflammatory positive feedback loop.

DAMPs released early after trauma (e.g., HMGB1, mitochondrial DNA) also rapidly induce the formation of neutrophil extracellular traps (NETs) [29,30]. These web-like structures, composed of decondensed chromatin and antimicrobial proteins, physically ensnare pathogens, thereby limiting infection [31,32]. However, when NETosis is excessive or clearance mechanisms fail, NETs become pathogenic. Their components (e.g., cell-free DNA, histones) function as potent DAMPs that damage vascular endothelium, capture platelets, express tissue factor, and induce immunothrombosis, amplifying the systemic inflammatory response [33,34,35,36,37,38,39].

### 2.3. Programmed Cell Death and Clearance of Neutrophils

The orderly resolution of inflammation and the initiation of tissue repair are critically dependent on the precise, timely clearance of spent neutrophils. This process hinges on programmed cell death (PCD) followed by efficient efferocytosis [40]; dysfunction in this axis prolongs inflammation and exacerbates tissue damage. 

In the complex milieu of trauma, neutrophils exhibit diverse death modalities. Apoptosis, a “silent” death characterized by cell shrinkage and membrane integrity, is the physiological default, enabling non-inflammatory removal [41,42,43]. However, the post-traumatic inflammatory environment (e.g., IL-8, TNF-α) [44] markedly suppresses apoptosis, extending neutrophil lifespan and permitting their continued release of damaging mediators [45]. In contrast, other death pathways are highly inflammatory. NETosis, a unique death pathway, releases the aforementioned pro-inflammatory NETs. Pyroptosis, a caspase-dependent lytic death triggered by inflammasome activation [46], is marked by membrane rupture and the release of potent cytokines IL-1β and IL-18 [47]. Ferroptosis, an iron-dependent form of lipid peroxidation, is an emerging pathway suggested to exacerbate damage as infiltrating neutrophils induce this process in resident cells [48,49,50]. 

Macrophage-mediated efferocytosis (the engulfment of apoptotic cells) is the rate-limiting step for inflammation resolution [51,52]. Apoptotic neutrophils externalize “eat-me” signals, such as phosphatidylserine (PS), which are recognized by macrophage receptors [53,54]. Critically, efferocytosis is not mere “garbage disposal”; it is an active signaling event that induces a phenotypic switch in the macrophage, from a pro-inflammatory (M1) to an anti-inflammatory, pro-repair (M2) state. This polarization is associated with the upregulation of IL-10, TGF-β, and specialized pro-resolving lipid mediators [55,56], which collectively suppress inflammatory cascades and promote tissue remodeling. Conversely, impaired efferocytosis leads to secondary necrosis of uncleared apoptotic cells, releasing their pro-inflammatory contents and reversing the resolution process. Thus, maintaining the integrity of the “death–clearance–repair” axis is fundamental to restoring homeostasis after trauma.

## 3. The Pathological Role of Neutrophils in Post-Traumatic Complications

### 3.1. Mechanisms Promoting Post-Traumatic Infection

#### 3.1.1. Neutrophil Dysfunction and Systemic Immunosuppression

Following severe trauma, the host often descends into a paradoxical state of “immunoparalysis” [57]. This state is characterized by a stark disconnect: while peripheral neutrophil counts (neutrophilia) are markedly elevated, their crucial anti-infective functions, including chemotaxis, phagocytosis, and bactericidal capacity, are profoundly compromised. This dysfunction is multifactorial. High circulating levels of granulocyte colony-stimulating factor (G-CSF) drive the bone marrow to release large quantities of functionally immature neutrophils [8,58]. Concurrently, the upregulation of programmed death-ligand 1 (PD-L1) on circulating neutrophils directly suppresses T-cell-mediated adaptive immunity [59]. This innate-adaptive crosstalk is further blunted by elevated systemic anti-inflammatory cytokines, such as IL-10, which induce a broader state of immune suppression [60]. Mounting evidence indicates that the downregulation of key activation markers on peripheral neutrophils (e.g., CD16) in severely injured patients correlates directly with the onset of secondary infections and poor outcomes [6,61], highlighting this phenotype as a potential biomarker for post-traumatic immune status.

#### 3.1.2. The Dual Effect of Neutrophil Extracellular Traps (NETs)

As previously discussed, NETs exhibit a stark duality in the context of post-traumatic infection. While their primary physiological role is protective, physically trapping and neutralizing bacteria, fungi, and viruses, their uncontrolled formation or impaired clearance becomes deeply pathogenic. NET components, particularly histones and cell-free DNA, function as potent DAMPs that directly inflict damage on vascular endothelium and epithelium, thereby undermining critical physical barriers. For instance, in acute lung injury (ALI/ARDS), excessive NETs promote microvascular thrombosis and induce alveolar epithelial cell death [62,63]. Similarly, in ischemia–reperfusion models, elevated intestinal NETs correlate with the loss of tight-junction proteins [64], a mechanism implicated in disrupting the mucosal barrier and facilitating lethal bacterial translocation. Furthermore, NETs create a toxic microenvironment that can polarize macrophages toward a pro-inflammatory M1 phenotype, activate inflammasomes, and induce pyroptosis, thereby amplifying the inflammatory cascade and exacerbating distant organ damage.

### 3.2. Mechanisms Driving Distant Organ Injury

#### 3.2.1. Neutrophil-Mediated Systemic Inflammatory Response

Hyper-activated neutrophils are the central executioners of the systemic inflammatory response syndrome (SIRS) that precedes MODS [8]. The overwhelming release of DAMPs following trauma systemically activates neutrophils, causing them to discharge a torrent of inflammatory mediators (e.g., TNF-α, IL-1β, IL-6) and cytotoxic effector molecules (e.g., ROS, NE). This payload not only inflicts direct damage on vascular endothelial and distant organ parenchymal cells but also recruits more immune cells, establishing a devastating positive feedback loop. The combined assault of ROS-mediated lipid peroxidation and NE-driven ECM degradation constitutes a primary upstream driver of endothelial barrier failure and progressive organ dysfunction [63,65].

#### 3.2.2. Immunothrombosis

Immunothrombosis defines the intricate crosstalk between the innate immune and coagulation systems [66]. Neutrophils, primarily via NETs, are central architects of this pathological interaction [67]. The anionic DNA scaffold of NETs provides a surface for activating coagulation factors [68]; NET-associated histones are potent platelet activators, and NETs promote the contact activation of Factor XII, amplifying the intrinsic coagulation cascade [69,70]. Furthermore, NE can enhance the pro-coagulant tendency by degrading endogenous anticoagulant molecules, such as tissue factor pathway inhibitor (TFPI) [67]. Physiologically, this response is adaptive, helping to “wall off” pathogens [66]. However, in severe trauma, this mechanism becomes catastrophically dysregulated, leading to widespread microvascular occlusion and severe tissue hypoperfusion [69,70]. This resultant microvascular occlusion and ischemic–hypoxic injury represent a key secondary mechanism aggravating distant organ dysfunction [71]. Consequently, targeting NET-driven coagulopathy has become a critical area of therapeutic investigation.

### 3.3. Inhibitory Effects of Neutrophils on Tissue Repair and Regeneration

Successful tissue repair is critically contingent upon an orderly, well-timed transition from a pro-inflammatory to a pro-repair phase. Neutrophils function as essential gatekeepers of this transition. While their early presence is vital for debridement, their persistent activation or delayed clearance is a primary barrier to successful regeneration. This inhibitory effect operates at two key levels: 

First, a sustained, pro-inflammatory microenvironment is directly cytotoxic to the regenerative process. The continuous release of ROS, NE, and NETs by persistently activated neutrophils inflicts severe bystander damage, degrading the provisional ECM and exhibiting direct cytotoxicity towards key reparative cells, including fibroblasts and endothelial progenitors. Furthermore, the chronic inflammatory milieu (rich in TNF-α) can suppress fibroblast collagen production [72], delaying granulation tissue formation and wound closure. In contexts such as bone repair, where spatiotemporal control is paramount, this uncontrolled inflammatory phase can severely damage osteogenic cells, impeding bone regeneration [73,74].

**Figure 1:**
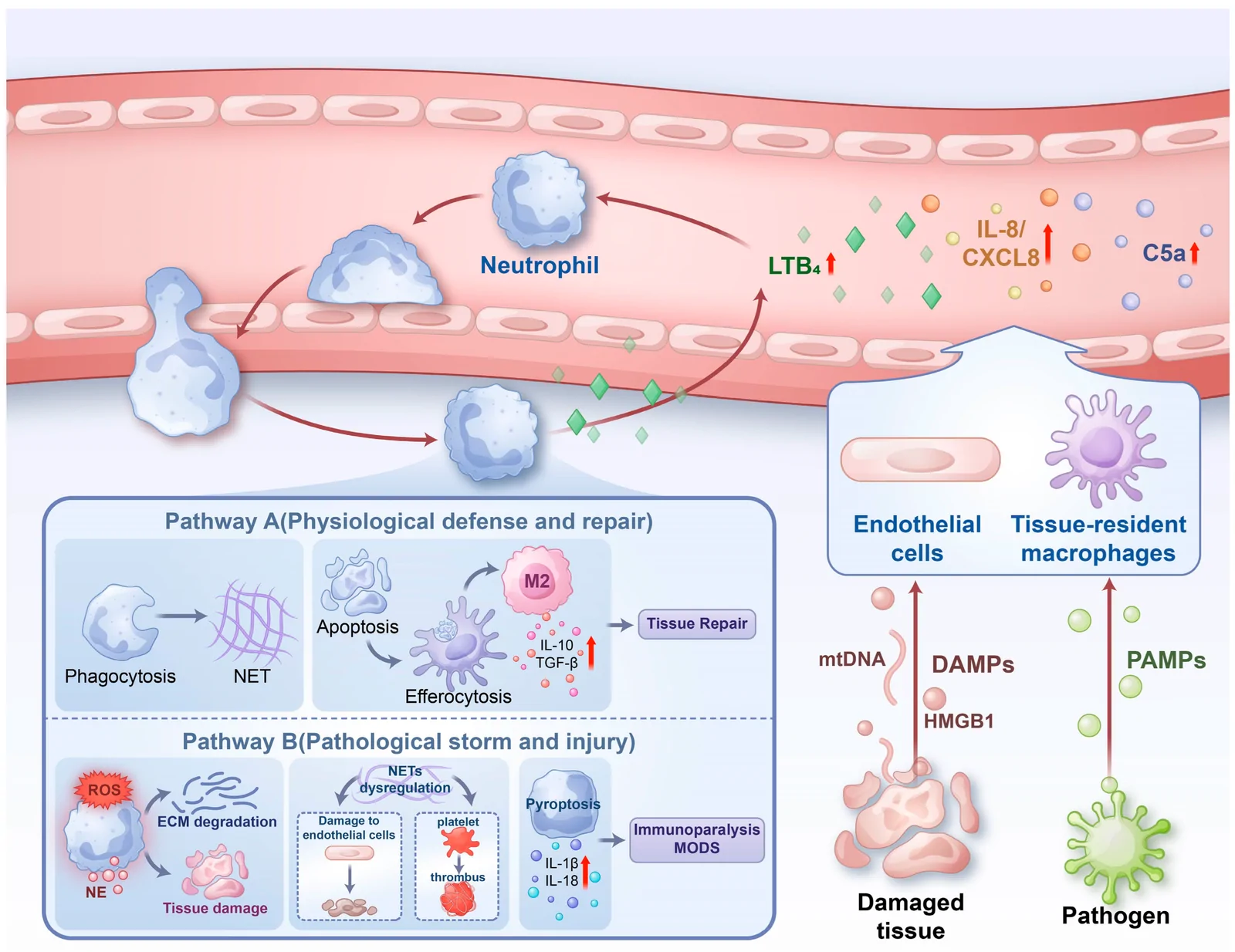
Neutrophil Recruitment and “Double-Edged Sword” Effects Post-Trauma. After trauma, damage-associated molecular patterns (DAMPs, e.g., HMGB1, mtDNA) and pathogen-associated molecular patterns (PAMPs) are released, which first activate tissue-resident macrophages and vascular endothelial cells to secrete chemokines (including IL-8, LTB4, and C5a); circulating neutrophils then infiltrate damaged tissues via the “rolling–adhesion–transendothelial migration” cascade, and during this process, early infiltrating neutrophils create a positive feedback loop by releasing LTB4 to further amplify neutrophil recruitment. This recruitment is followed by two distinct functional outcomes of neutrophils: in the physiological defense and repair pathway, neutrophils clear pathogens through phagocytosis and controlled formation of neutrophil extracellular traps, then undergo apoptosis; subsequent efferocytosis of apoptotic neutrophils by macrophages drives the latter toward a pro-repair M2 phenotype that secretes IL-10 and TGF-β, thereby promoting tissue regeneration. In contrast, in the pathological dysfunction and injury pathway, hyperactivated neutrophils release excessive reactive oxygen species and elastase, leading to extracellular matrix degradation and bystander tissue damage; uncontrolled NETs further induce immunothrombosis, while lytic cell death of neutrophils releases IL-1β and IL-18 to exacerbate the inflammatory response, ultimately resulting in immunoparalysis and multiple organ dysfunction syndrome This figure was created using Adobe Illustrator 2024 (Adobe Systems, San Jose, CA, USA).

Second, neutrophils indirectly sabotage repair by dictating macrophage polarization. In an ideal healing trajectory, apoptotic neutrophils are efficiently cleared by macrophages (efferocytosis), a process that actively triggers macrophage polarization to the pro-repair M2 phenotype [55,56]. However, in the pathological state of trauma where efferocytosis is often impaired, uncleared necrotic neutrophils and persistent NETs continue to release pro-inflammatory factors [75,76]. This environment actively skews macrophages toward the M1 phenotype and can trigger pyroptosis [29,77], thereby suppressing the M2-driven pro-resolving and regenerative programs essential for tissue restoration (Figure 1). 

The M2 phenotype secretes IL-10 and TGF-β, thereby promoting tissue regeneration [78]. In contrast, in the pathological dysfunction and injury pathway, hyperactivated neutrophils release excessive reactive oxygen species and elastase, leading to extracellular matrix (ECM) degradation and bystander tissue damage; uncontrolled NETs further induce immunothrombosis, while lytic cell death (such as pyroptosis) of neutrophils releases IL-1β and IL-18 to exacerbate the inflammatory response, ultimately resulting in immunoparalysis and multiple organ dysfunction syndrome [46,79].

## 4. Molecular Mechanisms Regulating Neutrophil Function

### 4.1. Transcriptional and Epigenetic Regulation

**Figure 2:**
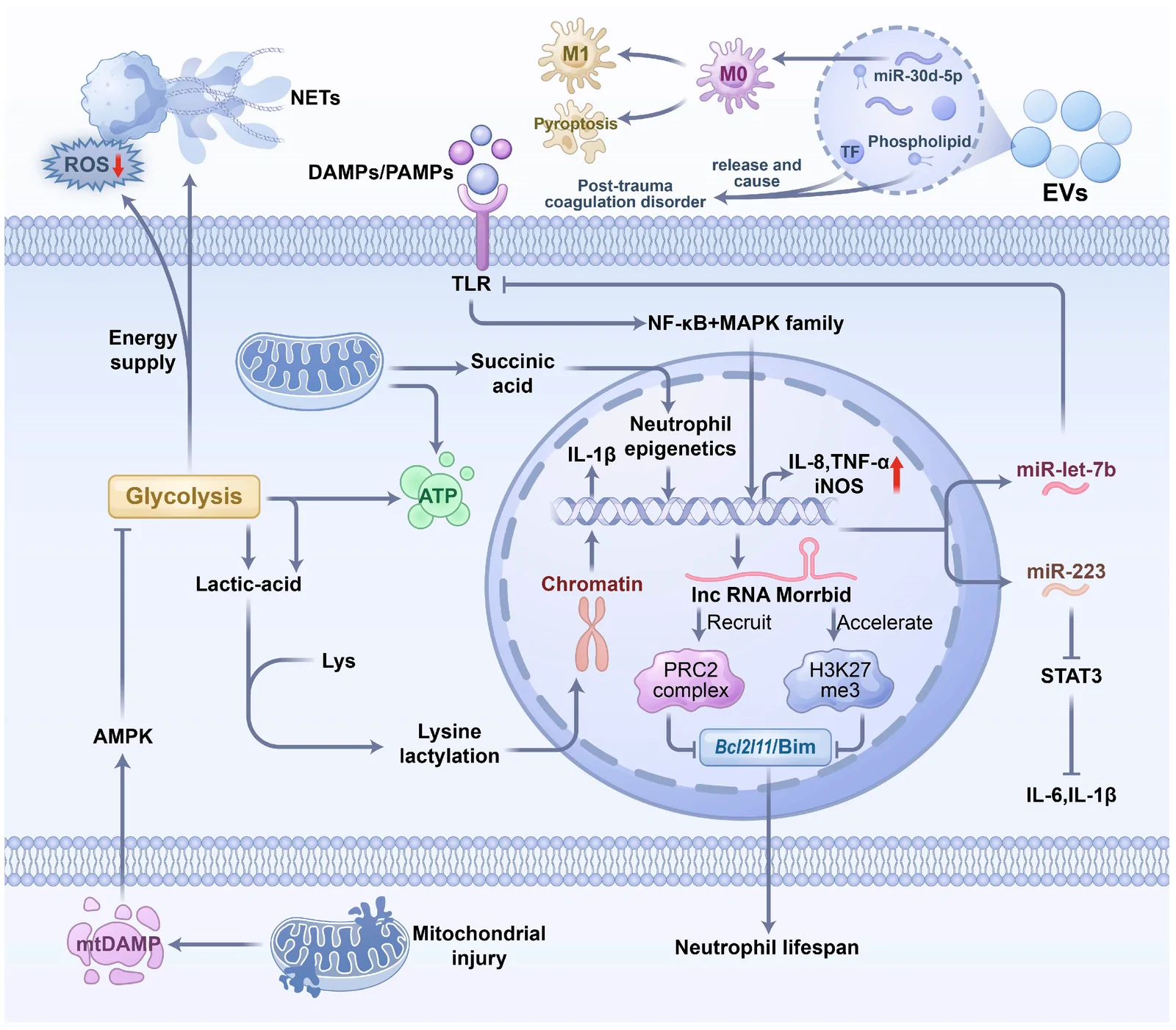
Key Regulatory Mechanisms of Neutrophils Post-Trauma. This figure illustrates neutrophil regulation across four dimensions: signal transduction, epigenetics, metabolic reprogramming, and intercellular communication. (1) Signal Transduction: TLRs recognize DAMPs/PAMPs, activating NF-κB and MAPK pathways to initiate pro-inflammatory gene transcription (e.g., IL-8, TNF-α). This is negatively regulated by miRNAs: miR-let-7b targets TLR4, while miR-223 inhibits the STAT3 axis to limit inflammatory factors. (2) Epigenetics: Nuclear lncRNA Morrbid recruits PRC2 to promote H3K27me3 deposition, repressing the pro-apoptotic gene *Bcl2l11* (Bim) and prolonging neutrophil lifespan. (3) Metabolic Reprogramming: Activated neutrophils rely on glycolysis. The resulting lactate alters local acidity and drives histone lactylation (Kla) to regulate inflammatory genes (e.g., IL-1β). Although mitochondria contribute minimally to energy, their metabolites (e.g., succinate) act as signaling molecules influencing the neutrophil epigenetic state. Conversely, mtDAMPs from damaged mitochondria activate AMPK, which inhibits glycolysis, ROS, and NETs, forming a negative feedback loop. (4) Intercellular Communication: Neutrophil-derived extracellular vesicles (EVs) act as messengers. EV-encapsulated miR-30d-5p promotes M1 macrophage polarization and pyroptosis. Furthermore, EVs carrying tissue factor (TF) contribute to post-traumatic coagulation disorders. This figure was created using Adobe Illustrator 2024 (Adobe Systems, San Jose, CA, USA).

Neutrophil effector functions are orchestrated by canonical signaling pathways and a complex network of non-coding RNAs. The nuclear factor-κB (NF-κB) and mitogen-activated protein kinase (MAPK) pathways represent the central hubs of acute activation. In trauma, DAMPs and PAMPs ligate pattern recognition receptors (e.g., Toll-like receptors), triggering downstream cascades [80,81] that rapidly activate NF-κB and MAPK family members (ERK, p38, JNK) [82]. This drives the transcription of a broad pro-inflammatory cache, including chemokines (e.g., IL-8/CXCL8), cytokines (e.g., TNF-α), and effector enzymes (e.g., iNOS) [83,84,85], thereby regulating recruitment, phagocytosis, and the oxidative burst (Figure 2). 

This core machinery is calibrated by non-coding RNAs, which act as critical “fine tuners” or rheostats. MicroRNAs (miRNAs) often function as “brakes” on inflammation. For instance, miR-let-7b directly targets TLR4 to suppress NF-κB overactivation [86,87], while the highly expressed miR-223 limits inflammation by targeting pathways such as STAT3 [88]. Concurrently, long non-coding RNAs (lncRNAs) influence neutrophil fate via epigenetic modulation. A compelling example is the lncRNA Morrbid, which recruits the PRC2 complex (containing EZH2) to promote local H3K27me3 deposition, thereby repressing the pro-apoptotic factor *Bcl2l11*/Bim. This axis, which precisely controls the lifespan of short-lived myeloid cells [89,90], represents a key molecular mechanism underlying the pathological persistence of neutrophils observed after severe trauma.

### 4.2. Intercellular Communication: The Role of Extracellular Vesicles

Neutrophils do not function in isolation; they actively “broadcast” their activation state systemically via extracellular vesicles (EVs), which are primarily classified into exosomes (30–150 nm, derived from the endosomal pathway) and microvesicles (100–1000 nm, shed directly from the plasma membrane). These EVs are laden with bioactive cargo, including miRNAs, proteins, and metabolites, that can reprogram recipient cell phenotypes [91,92]. This process disseminates inflammation far from the primary injury site. For example, neutrophil-derived EVs rich in miR-30d-5p have been shown to promote M1 macrophage polarization and induce pyroptosis upon uptake [93]. Furthermore, trauma increases circulating microvesicles that carry tissue factor (TF) and procoagulant phospholipids [94,95]. These EVs are not mere bystanders; they are direct effectors that propagate post-traumatic coagulopathy [96]. Thus, EV-mediated signaling constitutes a critical mechanism for the systemic amplification of local inflammation and pathology.

### 4.3. Metabolic Reprogramming

A recent paradigm shift in immunology is the recognition that metabolic reprogramming is not merely a consequence of immune activation but an active driver of cell function. Mature neutrophils, which rely almost exclusively on glycolysis [97], are a prime example. Upon activation, they dramatically increase their glycolytic rate (a Warburg-like effect) [98], which serves two purposes: rapid ATP generation for energy-intensive tasks (e.g., phagocytosis) and the production of metabolites that function as signaling molecules. 

The end-product lactate, long dismissed as a waste product, is now recognized as a critical signaling metabolite. It acidifies the microenvironment, acts as a ligand for the GPR81 (HCAR1) receptor, and, most critically, serves as the substrate for lysine lactylation (Kla). This novel post-translational histone modification directly links metabolic state to gene expression, creating a feedback loop that can regulate inflammatory gene expression (e.g., *IL1B*) [99,100,101]. 

While often considered vestigial for ATP production in mature neutrophils [97], mitochondria are crucial signaling hubs. They regulate chemotaxis, and their metabolites (e.g., succinate) can function as epigenetic signals [102]. Following severe trauma, circulating mitochondrial DAMPs (mtDAMPs) released from damaged tissues are recognized by neutrophils. This interaction triggers the phosphorylation and activation of AMP-activated protein kinase (AMPK), a central energy sensor. Activated AMPK suppresses glycolytic flux, lactate production, and subsequent ROS generation and NET formation. This mtDAMP-AMPK axis is proposed as a key negative feedback loop [103], providing a metabolic basis for the “low-reactive/tolerant” state that contributes to post-trauma immunoparalysis.

## 5. Clinical Significance and Therapeutic Strategies Targeting Neutrophils

### 5.1. Exploration of Neutrophils as Clinical Biomarkers

From Rudimentary Metrics to Functional Assessment: Historically, clinical assessment of inflammation has relied on rudimentary metrics, such as the absolute neutrophil count and derived ratios like the neutrophil/lymphocyte ratio (NLR) [104]. While numerous studies validate that an early post-traumatic neutrophilia correlates with subsequent MODS risk, these gross counts are insufficient. They fail to capture the complex functional state of the cells, critically, whether they are in a hyper-active, “primed” state or a dysfunctional, “immunoparalytic” state [8].

Development of High-Fidelity Molecular Markers: Current research is intensely focused on identifying molecular markers that reflect neutrophil functional status. NET-related products, including myeloperoxidase-DNA complexes (MPO-DNA) and citrullinated histone H3 (CitH3), are promising candidates [105,106]. Circulating cell-free DNA (cfDNA) is elevated after trauma but lacks specificity. In contrast, CitH3 is a specific biomarker of NET formation, and its prognostic value, while demonstrated in septic shock, requires urgent validation in the context of non-infectious, post-traumatic inflammation [107,108].

Precise Subtyping via Single-Cell Technologies: The cutting edge of biomarker discovery lies in single-cell technologies. Single-cell transcriptomic analyses of patient samples are dismantling the traditional view of neutrophils as a homogeneous group. These studies reveal substantial in vivo heterogeneity, identifying multiple, functionally distinct neutrophil subsets. The presence or relative abundance of specific subsets (e.g., specific pro-inflammatory or immunosuppressive phenotypes) correlates strongly with disease severity and outcomes [5,6]. This “deep phenotyping” at the single-cell level promises to transcend crude cell counts, enabling precise patient stratification and individualized prognostic assessment.

### 5.2. Therapeutic Strategies Targeting Neutrophils

Targeting NETs: A Therapeutic Dilemma: Given their profound pathological roles, NETs are a highly attractive therapeutic target. Strategies are twofold: promoting NET degradation (e.g., recombinant human DNase) and inhibiting NET formation (e.g., specific PAD4 inhibitors to block histone citrullination) [109,110]. However, the physiological role of NETs in host defense presents a formidable translational hurdle. The critical question remains: can these interventions suppress pathological NETosis without dangerously compromising the host’s ability to clear secondary infections? Balancing efficacy with safety is the key challenge to be resolved before clinical application.

Modulating Pathological Cell Death: The overactivation of lytic death pathways, particularly pyroptosis and necroptosis, is a major driver of post-traumatic inflammatory storms [111]. Consequently, specific small-molecule inhibitors (e.g., caspase inhibitors, RIPK inhibitors) that block these “self-destruct” pathways have shown significant organ-protective effects in pre-clinical sepsis models [112]. This strongly suggests that precisely suppressing pathological neutrophil necrosis in severe trauma could effectively mitigate secondary organ injury. Yet, this strategy faces a similar challenge to NET inhibition: weighing the potential impact on essential immune clearance mechanisms.

The Future: Precision Functional Modulation—Rather than ablating cells or completely blocking a function, the future of neutrophil-targeted therapy lies in nuanced modulation. This approach is exemplified by strategies targeting key chemokine axes, such as the IL-8/CXCR1/2 axis. By using anti-IL-8 monoclonal antibodies [113] or small-molecule CXCR2 antagonists [114], pre-clinical models show a marked reduction in neutrophil-mediated tissue damage [115]. This strategy does not “disarm” the neutrophil; it “re-calibrates” its response by specifically suppressing excessive recruitment and activation at injury sites. This approach offers a superior balance between mitigating inflammation and preserving antimicrobial capacity, representing a potential breakthrough for achieving precision immunotherapy in trauma.

## 6. Conclusions and Future Perspectives

Neutrophils are now understood as critical, plastic orchestrators of post-traumatic immunity, yet this same plasticity, which drives both host defense and secondary organ injury, remains a central therapeutic challenge. Our progress is fundamentally constrained by an incomplete understanding of their functional heterogeneity. This knowledge gap is perpetuated by two primary obstacles: a persistent “translational dilemma” in preclinical models, where ubiquitous murine systems poorly recapitulate human NET biology [116,117] and more faithful large animal models lack specific investigational tools, and an insufficient grasp of the “spatiotemporal dynamics” of neutrophil subsets, which exhibit complex, biphasic responses ranging from systemic hyporesponsiveness to potent localized hyperactivation within injured tissues [5,118,119]. 

Future breakthroughs will be driven by the convergence of high-resolution technologies designed to decipher this complexity. The “deep integration of single-cell and spatial omics” represents a critical frontier. Moving beyond simply cataloging subsets, this multi-modal approach will enable the in-situ mapping of specific neutrophil populations within the organ microarchitecture, revealing their precise anatomical niches, functional states, and the critical ligand–receptor networks they form. This high-resolution mapping will provide the most direct evidence for novel, subset-specific therapeutic targets.

Simultaneously, “Artificial Intelligence (AI)” must be leveraged to conquer the profound clinical heterogeneity of trauma. By integrating massive “multi-modal patient data” spanning from genomics to clinical phenotypes, researchers can use machine learning algorithms to establish high-precision prognostic systems and develop novel, immune-based patient stratification. Ultimately, this convergence of multi-omics and AI will shift trauma immunology from a “one-size-fits-all” paradigm to one of true precision. AI-driven models that process dynamic biomarkers—including circulating miRNA-enriched extracellular vesicles (miRNA-EVs) and metabolites—offer theoretical potential to predict the risk of severe complications, such as NET-mediated ARDS, and to guide the ‘optimal individualized timing’ of targeted interventions. Nevertheless, translating this potential into routine clinical practice will entail overcoming major barriers. Principal among these is the critical requirement for large-scale, multi-center prospective cohort studies to rigorously establish the predictive performance and clinical value of these models. In the absence of such stringent validation, their use will remain a conceptual vision rather than a practically applicable clinical tool.

## Data Availability

No new data were created or analyzed in this study. Data sharing is not applicable to this article.

## Abbreviations

The following abbreviations are used in this manuscript:

MODS	multiple organ dysfunction syndrome
ROS	reactive oxygen species
NETs	neutrophil extracellular traps
DAMPs	damage-associated molecular patterns
PAMPs	pathogen-associated molecular patterns
IL-8/CXCL8	interleukin-8/C-X-C motif chemokine ligand 8
CXCR1/2	C-X-C chemokine receptor 1/2
C5a	complement fragment C5a
LTB4	leukotriene B4
PSGL-1	P-selectin glycoprotein ligand-1
ICAM-1	intercellular adhesion molecule 1
NADPH	nicotinamide adenine dinucleotide phosphate
NE	neutrophil elastase
ECM	extracellular matrix
HMGB1	high mobility group box 1
PCD	programmed cell death
TNF-α	tumor necrosis factor-α
IL	interleukin
PS	phosphatidylserine
TGF-β	transforming growth factor-β
G-CSF	granulocyte colony-stimulating factor
PD-L1	programmed death-ligand 1
CD16	cluster of differentiation 16
ALI/ARDS	acute lung injury/acute respiratory distress syndrome
SIRS	systemic inflammatory response syndrome
TF	tissue factor
ATP	adenosine triphosphate
TFPI	tissue factor pathway inhibitor
NF-κB	nuclear factor-κB
MAPK	mitogen-activated protein kinase
ERK	extracellular signal-regulated kinase
JNK	c-Jun N-terminal kinase
iNOS	inducible nitric oxide synthase
TLR4	Toll-like receptor 4
STAT3	signal transducer and activator of transcription 3
lncRNAs	long non-coding RNAs
Morrbid	myeloid RNA regulator of Bim-induced death
PRC2	Polycomb repressive complex 2
EZH2	enhancer of zeste 2 polycomb repressive complex 2 subunit
H3K27me3	histone H3 lysine 27 trimethylation
*Bcl2l11*/Bim	BCL2-like 11/apoptosis regulator Bim
EVs	extracellular vesicles
AMPK	AMP-activated protein kinase
NLR	neutrophil/lymphocyte ratio
MPO-DNA	myeloperoxidase-DNA complexes
CitH3	citrullinated histone H3
cfDNA	cell-free DNA
DNase I	deoxyribonuclease I
PAD4	peptidylarginine deiminase 4
RIPK	receptor-interacting serine/threonine-protein kinase
AI	Artificial Intelligence
